# Accuracy of an Automated Plasma Apolipoprotein E4 Proteotyping Immunoassay for Determination of *APOE* Genotype

**DOI:** 10.1002/alz70856_107634

**Published:** 2026-01-09

**Authors:** Rachael Smith, Yara Alkhodair, Moones Yadegari, Mari L DeMarco

**Affiliations:** ^1^ Division of Neurology, Department of Medicine, University of British Columbia, Vancouver, BC, Canada; ^2^ Djavad Mowafaghian Centre of Brain Health, Vancouver, BC, Canada; ^3^ Department of Pathology and Laboratory Medicine, St. Paul's Hospital, Providence Health Care, Vancouver, BC, Canada; ^4^ Department of Pathology and Laboratory Medicine, University of British Columbia, Vancouver, BC, Canada

## Abstract

**Background:**

Anti‐amyloid therapies (AAT) for Alzheimer's disease are associated with the risk of amyloid‐related imaging abnormalities (ARIA). *APOE4* homozygotes (E4/E4) have the highest risk, followed by *APOE4* carriers (E4), making *APOE* genotyping essential for risk assessment. While *APOE4* allelic status is routinely determined via DNA (e.g., by RT‐PCR), recently developed automated immunoassays offer an alternative approach by measuring the apolipoprotein E4 (apoE4) concentration in plasma (i.e., proteotyping). In this study, we evaluated the diagnostic accuracy of an apoE4 proteotyping assay (Fujirebio Lumipulse) and its suitability for ARIA risk assessment.

**Method:**

This diagnostic accuracy study included 104 plasma samples from unique individuals with known *APOE* genotypes determined by RT‐PCR: E2/E3 = 8, E3/E3 = 45, E2/E4 = 1, E3/E4 = 44, and E4/E4 = 6. Plasma samples were analyzed using Fujirebio's Lumipulse G1200 system with the Lumipulse G ApoE4 and Pan‐ApoE assays. These assays measure apoE4 and total apoE protein concentrations, respectively, and their ratio is used to infer E4 allelic status (non‐E4, E4, or E4/E4). The assays were further evaluated for precision, potential interferences, and sample stability across freeze/thaw cycles.

**Result:**

The plasma proteotyping assay demonstrated 100% accuracy in distinguishing the presence or absence of an E4 allele compared to RT‐PCR. It correctly classified all non‐E4 individuals (*n* = 53), all E4/E4 (*n* = 6), and 41 of 45 E4 heterozygotes (4 heterozygotes were misclassified as E4/E4). The ApoE4 and Pan‐ApoE assays had total coefficients of variation of 8.5% and 4.0%, respectively. No significant interference was observed for hemolysate up to ∼5.25 g/L of hemoglobin or for lipemia up to ∼500 mg/dL of intralipid. Additionally, freeze‐thaw testing showed no significant impact on assay performance for up to 4 freeze/thaw cycles.

**Conclusion:**

The evaluated plasma apoE4 phenotyping assay demonstrated perfect accuracy in detecting the presence or absence of an E4 allele. However, it did not reliably distinguish E4 heterozygotes from homozygotes, with several heterozygotes misclassified as E4/E4. In the context of AAT treatment eligibility and ARIA risk assessment, proteotyping is an accurate method for both ruling in and out the presence of an E4 allele but E4/E4 results specifically should be confirmed via genotyping.

